# Emerging Roles of PRDM Factors in Stem Cells and Neuronal System: Cofactor Dependent Regulation of PRDM3/16 and FOG1/2 (Novel PRDM Factors)

**DOI:** 10.3390/cells9122603

**Published:** 2020-12-04

**Authors:** Paweł Leszczyński, Magdalena Śmiech, Emil Parvanov, Chisato Watanabe, Ken-ichi Mizutani, Hiroaki Taniguchi

**Affiliations:** 1 Department of Experimental Embryology, Laboratory for Genome Editing and Transcriptional Regulation, Institute of Genetics and Animal Biotechnology of the Polish Academy of Sciences, 05-552 Jastrzębiec, Poland; p.leszczynski@igbzpan.pl (P.L.); m.smiech@igbzpan.pl (M.Ś.); 2 Department of Mouse Molecular Genetics, Institute of Molecular Genetics of the Czech Academy of Science, 142 20 Vestec, Prague, Czech Republic; eparvanov@gmail.com; 3 Department of Stem Cells and Human Disease Models, Research Center for Animal Life Science, Shiga University of Medical Science, Shiga 520-2192, Japan; cwatanab@belle.shiga-med.ac.jp; 4 Laboratory of Stem Cell Biology, Graduate School of Pharmaceutical Sciences, Kobe Gakuin University, Kobe 650-8586, Japan; mizutani@pharm.kobegakuin.ac.jp

**Keywords:** PRDM, FOG, stem cells, neurons, NuRD, CtBP

## Abstract

PRDI-BF1 (positive regulatory domain I-binding factor 1) and RIZ1 (retinoblastoma protein-interacting zinc finger gene 1) (PR) homologous domain containing (PRDM) transcription factors are expressed in neuronal and stem cell systems, and they exert multiple functions in a spatiotemporal manner. Therefore, it is believed that PRDM factors cooperate with a number of protein partners to regulate a critical set of genes required for maintenance of stem cell self-renewal and differentiation through genetic and epigenetic mechanisms. In this review, we summarize recent findings about the expression of PRDM factors and function in stem cell and neuronal systems with a focus on cofactor-dependent regulation of PRDM3/16 and FOG1/2. We put special attention on summarizing the effects of the PRDM proteins interaction with chromatin modulators (NuRD complex and CtBPs) on the stem cell characteristic and neuronal differentiation. Although PRDM factors are known to possess intrinsic enzyme activity, our literature analysis suggests that cofactor-dependent regulation of PRDM3/16 and FOG1/2 is also one of the important mechanisms to orchestrate bidirectional target gene regulation. Therefore, determining stem cell and neuronal-specific cofactors will help better understanding of PRDM3/16 and FOG1/2-controlled stem cell maintenance and neuronal differentiation. Finally, we discuss the clinical aspect of these PRDM factors in different diseases including cancer. Overall, this review will help further sharpen our knowledge of the function of the PRDM3/16 and FOG1/2 with hopes to open new research fields related to these factors in stem cell biology and neuroscience.

## 1. Introduction

PRDI-BF1 (positive regulatory domain I-binding factor 1) and RIZ1 (retinoblastoma protein-interacting zinc finger gene 1) (PR) homologous-domain-containing (PRDM) transcription factors have received considerable attention recently due to their importance in regulating the development and function of various tissues and organ systems. The PRDM protein family is a group of 19 poorly studied factors that are involved in a wide range of cellular processes [1,2,3,4]. The PR domain is associated with the catalytic SET (suppressor of variegation 3–9, enhancer of zeste and trithorax) domain, which possesses histone lysine methyltransferase (HMT) activity [5]. Although some PRDM proteins have not been shown to have intrinsic HMTase activity [6,7,8], several studies have confirmed that PRDM2, PRDM3, PRDM8, PRDM9, and PRDM16 possess this capability [9,10,11,12,13,14]. 

**Figure 1:**
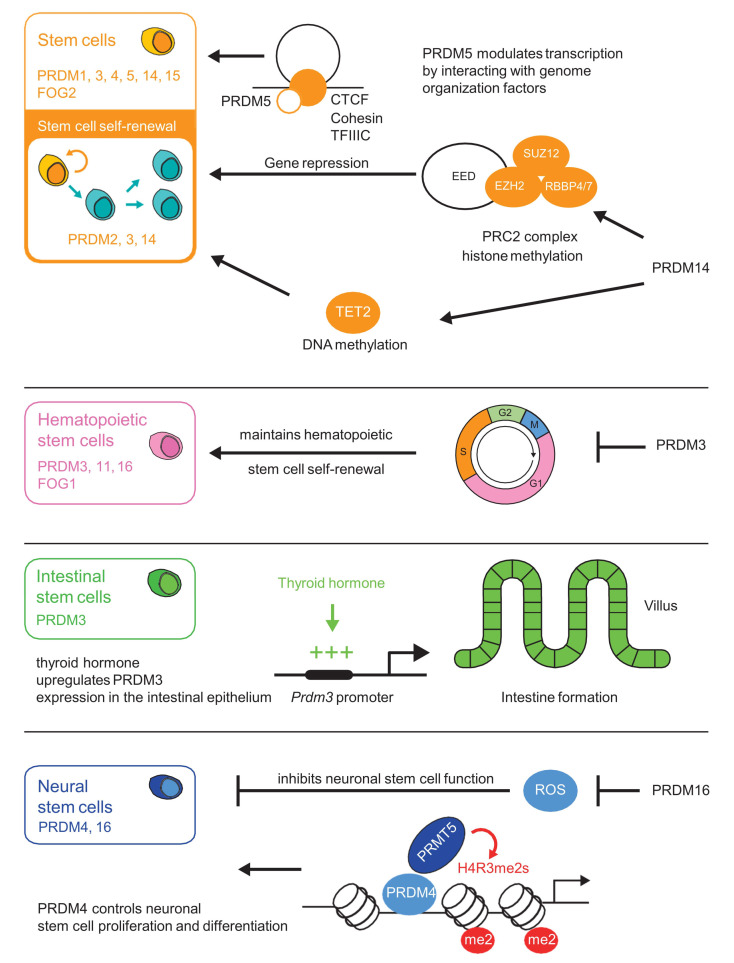
PRDI-BF1 (positive regulatory domain I-binding factor 1) and RIZ1 (retinoblastoma protein-interacting zinc finger gene 1) (PR) homologous domain containing (PRDM) factors play important roles in stem cell maintenance. PRDM3 and PRDM16 exhibit a crucial regulatory role in hematopoietic stem cell (HSC) and progenitor cell maintenance during fetal development [28,29,30,31,32,33]. PRDM1 determines the fate of embryonic stem cells and their progenitors [34,35]. PRDM14 plays an important role in governing the gene machinery responsible for maintaining the pluripotent state of embryonic stem cells. PRDM14 reprograms somatic cells to induce pluripotent stem cells through epigenetic pathways [36,37]. PRDM15 is also a transcriptional regulator of key genes involved in the maintenance of naive pluripotency of embryonic stem cells [38].

Depending on the cellular or tissue context, PRDM proteins mediate either transcriptional repression or activation. As several PRDM proteins appear to be enzymatically inactive, they achieve transcriptional regulation through interaction with transcription factors and histone-modifying enzymes. Interacting proteins include the Polycomb repressive complex 2 (PRC2), HMTs, histone acetyltransferases (HATs), histone deacetylases (HDACs), protein arginine N-methyltransferase 5 (PRMT5), and lysine-specific demethylase 1 (LSD1) [7,10,15,16,17,18,19,20]. For instance, the interplay between PRDM3 and the Suv39H1 HMT [21] leads to gene repression through H3K9 methylation. PRDM1, PRDM5, PRDM6 and PRDM12 are also known to interact with G9a HMT and repress gene expression through methylation of H3 lysine 9 [6,7,8,12,22]. PRDM proteins are involved in several developmental processes such as stem cell maintenance (Figure 1, Table 1), hematopoiesis, and adipogenesis [12,23]. Recent studies have highlighted the importance of these factors during neuronal development [24,25,26], including brain or spinal cord formation [26,27].

The PR domain is followed by repeated zinc fingers (proline-rich domains) mediating sequence- specific DNA binding and protein-protein interactions with other histone-modifying enzymes, and plays a role in nuclear import [23,64,65,66,67,68,69]. PRDM3 and PRDM16 display 63% nucleotide and 56% amino acid homology [70]. They exhibit intrinsic HMT activity towards histone 3 lysine 9 (H3K9), a mark typically associated with repressed transcription [9]. Other in vitro studies show that PRDM3 and 16 are involved in gene expression activation via methylation of histone 3 lysine 4 (H3K4) [9,24,71,72]. An additional pathway by which PRDM3 and PRDM16 govern gene expression through modification of chromatin structure is via the formation of protein complexes, such as the CtBP and NuRD [73,74,75]. Interestingly, FOG proteins, recently defined as PRDM factors, have also been confirmed to exert their function through the interaction with the NuRD complex and the CtBP protein [76,77,78], and it has been proposed that these factors may control cell fate decisions in stem cells and neuronal cells through the CtBP and NuRD complexes in a similar fashion to PRDM3 and PRDM16 [73,79]. In this review we will discuss the possible mechanism for their function in stem cell and neuronal systems through the interaction with their cofactors. 

## 2. PRDM Factors Are Substantial Players in Stem Cells

In recent years, the number of studies on the role of PRDM proteins in stem cells and cell differentiation has increased significantly. In this section we discuss studies on the role of PRDM proteins in maintaining self-renewal and pluripotency of stem cells, and in the neuronal system with potential molecular mechanisms that regulate the action of PRDM proteins. Highly pluripotent stem cells derived from the inner cell mass (ICM) are generated from precursors with high PRDM1 expression. Moreover, PRDM1-positive cells display a gene expression profile associated with the early specification of embryonic cells toward germ cell identity [35]. Additionally, the silencing of PRDM1 in human embryonic stem cells (hESCs) changes germline potential and directs cell differentiation towards neuronal specification by increasing SOX2 expression [80], suggesting that PRDM1 acts as a switch for neural or germline cell fate by inhibiting SOX2 expression during human development. The pluripotent state in stem cells can also be controlled by epigenetic regulation. PRDM14 is an important player regulating the epigenetic state and the transcription network in stem cells [56,81]. A genome-wide RNAi screen study revealed that PRDM14 colocalizes with stemness transcription factors such as OCT3/4, SOX2 and NANOG to maintain stem cell identity [37]. Moreover, it has been shown that the recruitment of OCT3/4 to the demethylated regulatory regions of pluripotency genes is driven by PRDM14 [36]. PRDM14-dependent pluripotency is mediated by reducing *Dnmt3a/b* and *Dnmt3l* expression and globally correlates with the CpG methylation landscape [55,82,83]. PRDM14 also maintains an active DNA demethylation status in the embryonic stem cells via TET (ten-eleven translocation) proteins [54]. These results suggest that the demethylation status observed during the induction of pluripotency is controlled by PRDM14. Besides PRDM14, PRDM15 was shown to be highly expressed in the embryo inner cell mass (ICM) [84] and plays a role as a safeguard of pluripotency in stem cells by regulating MAPK–ERK and WNT signaling [38]. Stimulation of the MAPK–ERK pathway triggers ESC lineage commitment [85], whereas the WNT pathway prevents differentiation of embryonic stem cells [86]. Stem cells with PRDM15 depletion showed a marked rise in nucleosome occupancy along with increased methylation and decreased acetylation at lysine 27 on histone H3 (H3K27ac to H3K27me3) at the promoter region of *Rspo1* (R-spondin 1) and *Spry1* (protein sprouty homolog 1), which are regulators of WNT signaling and MAPK–ERK pathways [38]. In order to maintain pluripotency and self-renewal of ESC, PRDM15 increases *Spry1* and *Rspo1* expression by decreasing nucleosome occupancy at the promoter sequence and allows RNA polymerase II recruitment [38].

## 3. An Overview of the Roles of PRDM Factors in the Neuronal System

**Figure 2:**
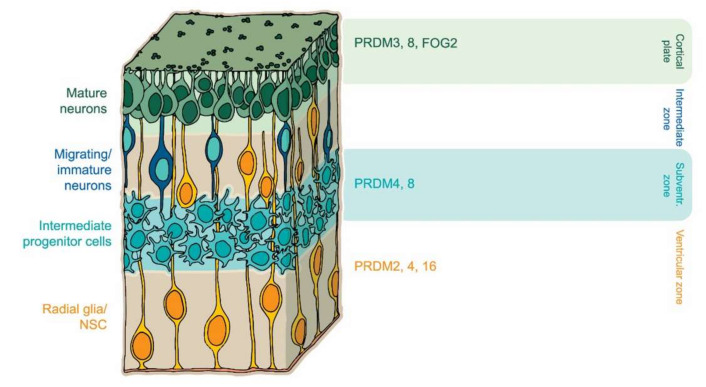
PRDM factors play multiple roles during central nervous system (CNS) development. PRDM16 controls the migration and differentiation of neuronal progenitors during cortical development [24,25]. FOG2 arranges a neuronal subtype identity [93] whereas PRDM8 controls axonal targeting [90].

Newly generated cortical neurons are derived from the division of radial glia progenitors in the ventricular zone (VZ). During neuronal maturation, progenitor cells move from VZ to the subventricular zone (SVZ) and then to the intermediate zone (IZ), where they adopt multipolar morphology (MP). In the subplate (SP) zone they acquire a bipolar shape and then settle in the cortical plate (CP) as mature neurons with defined characteristics [87,88]. Role of the PRDM factors and their expression in central nervous system (CNS) is depicted in Figure 2, Table 2. PRDM8 was shown to play a role in the development of brain structures. High expression of PRDM8 is found in the upper part of the IZ where it regulates the transition from multipolar to bipolar morphology of cortical neurons [89]. Moreover, mice carrying a *Prdm8* gene deletion displayed a significant reduction of brain growth along with a decreased number of neocortical neurons, indicating its essential role in neocortical development [26]. It has also been shown that basic helix-loop-helix family member E22 (BHLHB5), another cofactor of PRDM8, can significantly influence the formation of neuronal plasticity. PRDM8 and BHLHB5 form a repressor complex that orchestrates the neuronal circuit [90]. Mice with PRDM8 or BHLHB5 deficiency display highly similar axonal mistargeting and behavioral abnormalities [90], indicating the inherent spatiotemporal role of these factors in the formation of the nervous system. Much like their functions in stem cell maintenance, PRDM proteins tune progenitors of neuronal proliferation and differentiation through epigenetic modifications. For instance, PRDM4 is part of an epigenetic complex regulating the proliferative capacity and modulating cell cycle progression in neural stem cells (NSCs). PRDM4 interacts with PRMT5 through the PR/SET domain and the latter modifies chromatin structure by symmetric dimethylation on arginine 3 of histone H4 (H4R3me2s), specifically in the undifferentiated NSCs. Furthermore, a decrease in PRDM4 expression in NSC demonstrated precocious neurogenesis [50]. 

It is interesting to note that PRDM12 is involved in the development of nociceptors. Mutation in human *PRDM12* has been found in patients with congenital pain insensitivity (CIP) [91]. This mutation was found to be located in His289 of PRDM12 and it disrupts the interaction between PRDM12 and HMT G9a (EHMT2). PRDM12 has been reported to interact with G9a (EHMT2) and leads to dimethylation of histone H3 on lysine 9 (H3K9me2) in P19 cells [22]. On the other hand, it was shown that PRDM12 ablation negatively affects the Ngn1/2-TrkA pathway which interferes with nociceptor maturation [92]. These observations suggest that PRDM12 functions through epigenetic mechanisms and could serve as a molecular target in the therapeutic treatment of pain.

## 4. The Function of PRDM3 and 16 in Stem Cells

Among the PRDM factors, emerging roles of PRDM3 and PRDM16 in diverse systems, including stem cells and the neuronal system, have been proposed [25,33,46,103]. Mouse embryos with *Prdm3* deletion exhibit various developmental defects and caused lethality at 10.5 days post coitum [100]. This is mainly attributed to impaired hematopoietic stem cell (HSC) proliferation. PRDM3 regulates hematopoietic stem cell proliferation by activating the *Gata2* promoter [46], and *Gata2* gene expression is greatly reduced in *Prdm3* mutant mice [100]. Interestingly, mice lacking GATA2 expression showed a phenotype similar to PRDM3-deficient embryos. This suggests that these factors cooperate in HSC gene regulation [46,116]. Moreover, PRDM3 plays important roles in long-term HSC self-renewal [45]. These findings suggest that PRDM3 is one of the major regulators of *GATA2* gene expression and that it is also important for HSC maintenance. In *Xenopus tropicalis*, Prdm3 is required for adult intestinal formation and for the maintenance of epithelial stem cell proliferation [28]. PRDM3 has been reported as a histone 3 lysine 9 mono-methyltransferase (H3K9me1) in mammals and controls heterochromatin integrity [9]. In zebrafish, Prdm3 controls the chromatin landscape by influencing H3K9me3 and H3K4me3 marks. Furthermore, Prdm3-deficient zebrafish embryos display a 40% reduction in H3K4me3 marks [117]. These findings imply that PRDM3 plays a crucial role in stem cell self-renewal and differentiation through histone methylation. Further investigation to elucidate how PRDM3 impacts global methylation patterns to regulate the stem cell state is warranted.

Much like *Prdm3*, *Prdm16* expression is required for HSC maintenance [32,33]. PRDM16 is also responsible for many developmental processes in brown fat tissue [118,119], heart [120] and craniofacial formation [121,122,123]. *Prdm16* deletion in mice induces dysregulation of HSC renewal and increases apoptosis [32]. Moreover, mutation of *Prdm16* causes a disturbance in gene expression related to hematopoietic stem cell function [32]. As mentioned previously, studies in mouse embryonic fibroblasts have identified that PRDM3 is an H3K9me1 methyltransferase. PRDM16 displays similar characteristics to PRDM3. Both proteins methylate histone H3 in the cytoplasm. SUV39H1 and SUV39H2 enzymes then convert H3K9me1 to H3K9me3 in the nucleus. These modifications reinforce the heterochromatin to be assembled in pericentric DNA and the nuclear lamina [9]. The integrity of heterochromatin is important for spatial genome organization and gene expression programs [9]. Thus, PRDM16 could be involved in the modification of epigenetic markers, thereby regulating the differentiation of stem cells and progenitor cells. Indeed, this phenomenon is observed in a myoblast differentiation model, suggesting a significant role for PRDM16 in cellular transformation. Ectopic overexpression of *Prdm16* reprograms C2C12 myoblasts into brown fat cells [119]. Direct reprogramming induced by PRDM16 is accompanied by hypermethylation of myogenin and *MyoD* promoters [124]. Taken together, PRDM3 and PRDM16 both play important roles in stem cell maintenance in several systems through epigenetic mechanisms. 

## 5. A Novel PRDM Factors, Friend of GATA (FOG) and its Function in Stem Cells

Recent studies have revealed that FOG1 is a PRDM family member [125]. FOG1/2 and PRDM3/16, carry a CtBP-binding sequence in their protein structure and repress transcription. Both proteins also contain a PR domain [64,125]. Historically, it was known that FOG1 regulates GATA1 transcription factor function and FOG2 governs GATA2 function. The interaction between the FOG family and GATA transcription factors is crucial in various tissues [126,127,128,129] where FOG proteins repress the transcriptional activity of GATA factors. Serious problems, such as failure in heart development, occur as a result of blocking the interaction between GATA4 and FOG2 [130]. Moreover, mutations in FOG2 hinder integration with GATA4 leading to congenital heart disease [131,132]. While FOG2 interacts with GATA4-6, little is known about its role in stem cells and the nervous system. Interestingly, much like AML1-PRDM3 [133], the AML1-FOG2 fusion protein have implications in myelodysplasia [134]. In this regard, the FOG2 protein may also regulate hematopoietic stem cell function. Human bone marrow mesenchymal stem cells (BM-MSC) are a heterogeneous population and only some have cardiomyogenic potential. BM-MSC subpopulations with high cardiomyogenic potential display high FOG2 gene expression [63]. This suggests that FOG2 is involved in cardiomyocyte progenitor cell function. Moreover, since the FOG family’s major role is to inhibit GATA factors, FOGs may be involved in regulatory processes in stem cells and progenitors in which GATA factors play major roles. 

It is known that *Fog1* deficiency is lethal for mice. Mouse embryos with this deficiency die between days 10.5 and 12.5 of gestation [135]. In these mice, erythropoiesis is highly disrupted and megakaryocytes are absent. Intriguingly, GATA1 acts as a hematopoietic transcription factor that induces erythrocyte and megakaryocyte differentiation. These findings suggest similar phenotypes in FOG1 and GATA1-deficiencies. Another study has clearly shown that GATA1 and FOG1 interaction is essential to promote megakaryocyte/erythrocyte lineage differentiation [135]. Moreover, FOG1 is considered a reprogramming factor that stimulates the stemness state in differentiated cells. In avian eosinophils, FOG1 overexpression leads to the dedifferentiation and generation of multipotent cells [136]. Furthermore, overexpression of *Fog1* in mouse hematopoietic lineages resulted in a decreased number of eosinophils [137]. Thus, FOG1 is likely an important factor that controls the stem cell state and its function is tightly associated with GATA1.

## 6. The Function of PRDM3 and PRDM16 in Neuronal Cells

In *Caenorhabditis elegans*, it has been suggested that *Egl-43* (the PRDM3 and PRDM16 orthologue in *C. elegans*) has a significant influence on nervous system development. During embryonic growth, two serotonergic hermaphrodite specific neurons migrate from the caudal position to the central part of the body. Disruption of *Egl-43* gene expression stops neuronal migration and further development [99]. Follow-up reports in higher organisms highlighted the importance of PRDM3 in the formation of neuronal identity. The cellular specification of olfactory receptor neurons in *Drosophila melanogaster* is coordinated by a context-dependent response to Notch signaling. Hamlet (the PRDM3 and PRDM16 orthologue in *Drosophila*) mediates this pathway and contributes to the development of a specific type of neuron [103]. During the initiation of olfactory receptor neuron (ORN) development, Hamlet proteins erase the Notch-active state in differentiating cells. This phenomenon provides new insights into the Notch-dependent signaling pathway. The activity of Hamlet protein influences gene expression by altering the methylation profiles at promoter regions, histone packing density and chromatin organization. Hamlet alters chromatin accessibility by enabling Su(H) (suppressor of hairless protein) binding to Notch-specific enhancers. Mouse embryos with *Prdm3* deletion displayed severe defects in nervous system development, but detailed studies on brain structures were not conducted [100]. In mammals, *Prdm3* transcription is strongly activated by retinoic acid (RA) in murine embryonal carcinoma P19 cells [101]. Moreover, *Prdm3* gene expression is upregulated in NSCs compared to human embryonic stem cells [138]. Additionally, ectopic overexpression of *Prdm3* induces neurogenesis in P19 cells without RA stimulation [101]. However, the neuronal-specific role of PRDM3 remains to be addressed in mammals, and PRDM3 could be implicated in the onset of neurogenesis. Recently, our study showed that P19 cells with *Prdm3* gene knock-out displayed earlier maturation of neurons along with the rapid proliferation of non-neuronal cells [102]. These findings strongly showed the significant role of PRDM3 in the formation of the mammalian nervous system in vivo. Chromatin structure and epigenetic modifications have been reported to be crucial for regulating gene expression during brain development [139]. In this regard, PRDM3 plays a role in the formation of synaptic plasticity via epigenetic regulation of gene transcription. Investigation of synaptic plasticity using an in vitro model demonstrated that PRDM3 is expressed in the nucleus of hippocampal neurons and may be implicated in neuronal activity associated with α-amino-3-hydroxy-5-methyl-4-isoxazolepropionic acid receptor (AMPAR) regulation [104]. miR-124 expression has been strongly associated with a homeostatic response during neuronal activity [104]. Interestingly, miR-124 transcription is directly dependent on the active complex of PRDM3 and HDAC1. Regulation of miR-124 expression in hippocampal and cortical neurons is partially explained by PRDM3-dependent reduction of mir-124 promoter activity [104]. It is known that gene expression in neurons highly depends on chromatin remodeling factors [140]. Therefore, the PRDM3–HDAC1 complex could be part of a bigger, specific chromatin remodeling complex involved in the fine-tuning of synaptic plasticity. PRDM3 activates genes associated with the self-renewal mechanism in hematopoietic stem cells in mouse myelodysplastic syndrome through an increase in methylation level at the miR-124 promoter [141]. As such, the relationship between PRDM3 and miR-124 is clearly supported in two different cellular models. Therefore, further studies are needed to determine whether PRDM3 directly affects the promoter status of other target genes in the neuronal system. 

**Figure 3:**
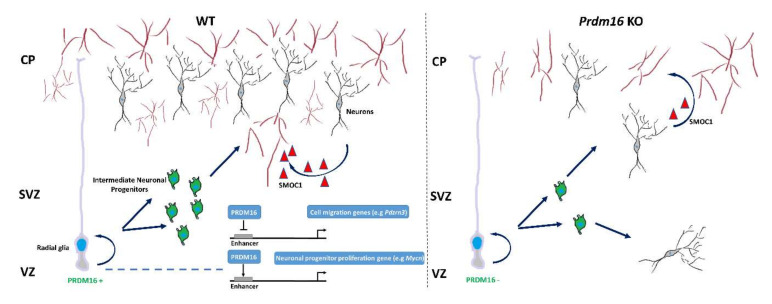
PRDM16 orchestrates neuronal migration and angiogenesis in the developing brain. PRDM16 organizes the migration of cortical neurons through enhancer-dependent silencing of *Pdzrn3* gene expression. In addition, PRDM16 controls the expression of genes associated with the amplification of neuronal progenitors (e.g., *Mycn*) [24]. PRDM16 positively regulates neuronal angiogenesis by enhancing the TGF-β signaling pathway via neuronally secreted SMOC1 [114]. VZ (ventricular zone), SVZ (subventricular zone), CP (cortical plate).

In the developing nervous system, PRDM16 is expressed in the SVZ of the neocortex and is crucial for neural stem cell maintenance. PRDM16 is a key player in maintaining neural stem cell and progenitor cells in the brain by controlling their temporal and spatial gene regulation networks [31]. In the mouse embryo, PRDM16 is mainly expressed in the VZ and SVZ but its expression decreases during brain maturation [114]. A conditional *Prdm16* deletion exhibits significant depletion of mouse neural stem cells in the SVZ as well as a limited ability to form self-renewing neurospheres in vitro [31]. Moreover, reduced *Prdm16* expression and complementary upregulation of the proneural gene *NeuroD1* in embryonic neural stem cells is crucial for the regulation of peroxisome proliferative activated receptor 1 (PGC1)-mediated changes in reactive oxygen species (ROS) levels, and this mechanism has been suggested to be important for neural migration in the developing cortex [25]. On the other hand, PRDM16 is preferentially expressed in adult neural stem cells and is required for their maintenance, partly by suppressing oxidative stress through the promotion of hepatocyte growth factor (HGF) expression [33]. Another study revealed that PRDM16 is involved in the migration and differentiation of neurons during embryonic cerebral cortex development [24]. Here, it was shown that epigenetic status during the early stages of neuronal differentiation is closely related to PRDM16-dependent mechanisms. Interestingly, the association of gene expression level with H3K27ac modification in radial cells (PRDM16 positive) is also found in mature cortical neurons where PRDM16 is not expressed. This phenomenon suggests that the previously acquired epigenetic memory remains during cortical neuronal development [24]. It has been suggested that the determination of neuronal position in the upper layer of the cerebral cortex is controlled by PRDM16-mediated repression of the gene encoding E3 ubiquitin ligase PDZ domain-containing RING finger protein 3 (PDZRN3). PRDM16 significantly reduces PDZRN3 expression in brain progenitor cells. This can be partially explained by the reduction in H3K27ac levels at the enhancer and promoter regions of *Pdzrn3*. Conversely, a significant increase in H3K27ac levels is observed in cortical cells in the absence of PRDM16. Impaired PRDM16 expression causes a significant increase in PDZRN3 expression in newly formed neurons, but the consequence of this genetic deregulation is the decreased ability of these cells to migrate to the upper brain layers. Moreover, a lack of PRDM16 in neuronal progenitors leads to abnormal dendritic morphology of mature neurons [24]. *Prdm16* deletion in neuronal stem cells causes dysregulation of angiogenesis. It was found that neurovascular communication depends in part on SMOC1, which is secreted by certain types of neurons. Neuronal SMOC1 interacts with TGFBR1 and activates the TGF-β-SMAD signaling pathway in endothelial cells. The loss of PRDM16 in neural progenitor disrupts this process and significantly impairs vascular growth in the developing brain [114]. The PRDM16 influence on neuronal cell migration and angiogenesis during central nervous system development is illustrated in Figure 3. Interestingly, RNA sequencing data show that disabling PRDM16 in progenitor cells (radial glia) resulted in a seven-fold increase in *Prdm3* expression [24]. Therefore, crosstalk between PRDM16 and PRDM3 during neuronal differentiation merits further investigation. Taken together, although functional studies in the neuronal system have just started, PRDM3 and PRDM16 most likely play paramount roles in neuronal cell fate decision and function. 

## 7. The Function of FOG1 and FOG2 in Neuronal Cells

FOG1 expression was found in the mouse mid-brain [129] as well as in the *Danio rerio* developing brain [142]. FOG1 affects the heart ventricular wall structure by regulating cardiomyocyte proliferation via the neuregulin (NRG)-ErbB-dependent signaling pathway [143]. NRG is a growth factor involved in the stimulation of ErbB tyrosine kinase receptors and thus affects cell survival, proliferation and differentiation in neuronal and non-neuronal systems. The NRG-ErbB axis is associated with susceptibility to mental illnesses such as bipolar disorder and schizophrenia [144,145]. A tight relationship between disease state and the NRG-ErbB pathway is indicated by the significant dysregulation of synaptic plasticity and neurotransmission (reviewed in [146]). Thus, the FOG1-controlled NRG-ErbB pathway may theoretically be involved in the development of synaptic plasticity and neuronal identity. However, due to the limited number of reports, the role of FOG1 in the nervous system development remains to be clarified. 

In the developing brain, FOG-2 expression is detectable from around day 10 of embryonic development (E10.5) [147]. Recent studies have reported that FOG2 may be involved in shaping neuronal identity [93]. FOG2 was found exclusively in postmitotic neurons in the cortex as well as in the thalamic reticular nucleus, the hippocampus, the amygdala and the hypothalamus. These findings strongly suggest that FOG2 plays a role as a transcriptional regulator during the final stage of neuronal maturation [93]. In addition, high expression of GATA transcription factors (e.g., GATA4 and GATA6) was found within the central nervous system in mice and humans, and this expression was mainly observed in post-mitotic neurons of the cerebral cortex and the hippocampus [148,149]. Nevertheless, the function of GATA proteins in shaping the identity of neurons has not been sufficiently investigated. GATA-dependent mechanisms that form neuronal identity could be partly explained through interaction with FOG1 and FOG2 proteins. FOG2, in cooperation with GATA6, significantly increases promoter activity of the *Kv4.2* (KCND2) gene (voltage-dependent potassium channels) in PC12 neuron-like cells. This study highlighted the importance of FOG2 in neuronal function and plasticity [115]. Tying FOG2-GATA6 to the regulation of voltage-gated ion channels could reveal new avenues in investigating the regulation of brain neuroplasticity and thus the formation of memory. Accurate tuning of gene expression during cortical development requires precise regulation of molecular machinery. In this context, FOG2 appears to be a mediator in the process of generating final cellular identities in the brain. FOG2 is involved in the control of corticothalamic projection neuron (CThPN) identity and positioning [93]. CThPNs are a diverse set of neurons that are important for the function of cortical circuitry. They are responsible for the access of sensory information to the cerebral cortex by modulating thalamic activity [150,151]. Interestingly, regulation of CThPN projection is controlled by the FOG2 and GATA4 complex via *Ctip2* (Coup-Tf interacting protein 1) promoter activity [93]. CTIP2 belongs to a group of factors crucial in postnatal brain development [152]. CTIP2 is also involved in the differentiation of postmitotic neurons and thus in memory and learning [152]. In humans, FOG2 mutations have been recognized primarily in congenital heart disease, but neurological and behavioral abnormalities have also been observed. Patients with a FOG2 deletion exhibit delayed or impaired speech ability, intellectual disability and seizures [153,154]. Based on the above information, it is postulated that FOG2 could be a crucial factor during the assembly of neural circuits and the acquisition of identities in postmitotic neurons. Further research is needed to determine the FOG2 role in brain development and neuronal plasticity.

## 8. NuRD Interacts with PRDM3, PRDM16, FOG1, and FOG2

**Figure 4:**
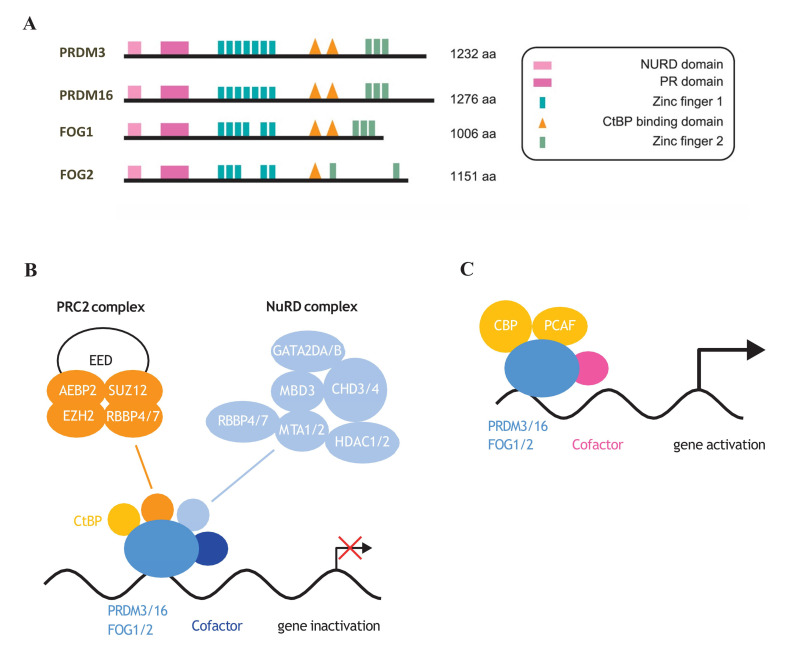
Protein structure of NuRD complex-dependent PRDM factors. (**A**) PR domain, CtBP binding sites, zinc fingers as well as NuRD complex binding elements are indicated. (**B**) PRDM3/16 and FOG1/2 by interaction with CtBP or NuRD complex negatively regulate gene expression during cellular differentiation [15,61,74,167,168]. (**C**) CBP- and PCAF-mediated acetylation of PRDM3 [169,170] increases its transcriptional activity [168].

Chromatin remodeling complex NuRD (nucleosome remodeling and deacetylase) primarily exhibits histone deacetylase activity and, therefore, exerts a repressor function [155,156]. The base composition of NuRD includes the metastasis-associated proteins MTA1/2/3, the histone deacetylases HDAC1 and HDAC2, the methyl-CpG-binding domain proteins (MBD2 or MBD3), CHD4, and the histone binding proteins RBBP4/7 [157,158,159]. NuRD regulates gene transcription associated with pluripotency and mediates the cellular response to differentiation signals in mouse embryonic stem cells (ESCs) [160,161]. NuRD activity has been reported to mediate the reduction of H3K27 acetylation facilitating recruitment of PRC2 and subsequent trimethylation of H3K27 in NuRD-dependent promoters [160,161]. The NuRD/MBD3 complex significantly shapes the final developmental stages of the brain. Despite high *Mbd3* expression in neuroepithelial cells (NECs) of the embryonic cortex [162], NuRD/MBD3 has been shown to be particularly important in regulating cell differentiation during neuronal specification [163]. Moreover, the NuRD complex affects synaptic plasticity in the mammalian brain and controls cortical neuron identity [140,164]. A good example of how NuRD influences the shape of terminal neuronal differentiation is by the regulation of the *Satb2*-*Ctip2* axis. CTIP2 and SATB2 are key transcription factors that define the development of two classes of projection neurons. *Ctip2* expression is terminated by the cooperation of SATB2 and NuRD. This, in turn, induces NuRD recruitment of HDAC1 and finally deacetylation of the *Ctip2* locus. Decreased levels of CTIP2 lead to the formation of a different subclass of projection neuron [165]. Recent studies have demonstrated that both PRDM3 and PRDM16 proteins interact with the chromatin remodeling NuRD complex through the RBBP4 (RB binding protein 4, chromatin remodeling factor; also known as NURF55) protein [73]. RBBP4 is recognized as a mediator that facilitates the association of chromatin with the NuRD complex by binding to histone H3 tails [166]. Amino acids from the N-terminus of PRDM3 and PRDM16 are responsible for binding to RBBP4 [73]. Interestingly, it has been reported that FOG1 and FOG2 also interact with the NuRD complex through their N-terminal amino acid sequence (Figure 4A,B) [76,78]. The N-terminal amino acid residues that interact with NuRD are conserved between PRDM3 and PRDM16 (Figure 4A). The first 12 residues of both proteins show high sequence similarity with histone 3 N-terminal residues [73]. It is known that RBBP4 interacts with LHX2 and regulates the expression of the *Sox11* and *Fezf2* genes; the most important factors determining the identity of neuronal subtype in the mouse cortex [164]. Since PRDM3 and PRDM16 interact with RBBP4 and the NuRD complex, these proteins may play a regulatory role in shaping the identity of neurons and their position in various brain structures. Moreover, since CtBP is also a major PRDM3 and PRDM16 modulator, and controls cell fate decisions (Figure 4B) [74], investigation is needed into how PRDM3 and PRDM16 select their binding to CtBP and NuRD complex.

FOG1/GATA1-dependent transcriptional repression is mediated by the NuRD complex. FOG-1 binds to NuRD via a 12-amino acid N-terminal motif [171]. FOG1 forms an active complex with NuRD to promote hematopoiesis. Depending on the composition of the protein complex, it also regulates cell lineage specification [61,171]. The FOG1/NuRD complex acts as a repressor of GATA1 and GATA2. This repression induces hematopoiesis by inhibiting GATA factors and subsequently halts mast cell differentiation [61]. Similarly, FOG2 modulates cardiomyocyte proliferation and heart morphogenesis by interacting with GATA4 to reduce GATA4-dependent gene expression [167,172]. However, the specific role of FOG2/NuRD interaction remains unknown.

*PRDM* genes can generate alternative forms of transcripts, which mainly include an isoform without a PR domain and a long product with a PR domain at the N-terminus. These two forms of transcripts can be generated using different promoter sites or by alternative splicing [64,65,67]. It is striking that the PRDM short product almost always acts as an oncogene and the long-form acts as a tumor suppressor [64,65]. PRDM3 is generated by combining two distinct genes- *MDS1* and *EVI1*. The construct without the PR domain is transcribed from one locus and is called EVI1 or sPRDM3 (short PRDM3) [173]. Mutations in the PRDM3 gene are common in acute myeloid leukemia and are related to reduced overall survival [174,175,176]. High expression of short PRDM3 is relatively often observed in myeloid or solid tumors. Intriguingly, the PR domain in PRDM3 appears to play a tumor suppressor function [177,178,179]. In addition, sPRDM3 appears to be frequently mutated in skin melanomas, colon, lung, bladder and endometrial cancers with simultaneous decreased expression of the long PRDM3 form [180]. Moreover, deficiency of the long version of PRDM3 also leads to a decrease in the number of hemopoietic stem cells and a loss of long-term repopulation capacity through deregulation of the p57-kip2 pathway [45]. Apparently, the PR domain of the PRDM16 protein plays a significant role in regulating gene expression through modification of epigenetic signatures. Loss of the PR domain of PRDM16 leads to a reduction in histone H3 acetylation (H3ac), H3K4me3 and H3K27me3 modifications at the *Pparγ* promoter. These changes attenuate the potential of adipogenic transdifferentiation in C2C12 myoblasts [124]. Furthermore, the isoforms of PRDM16 show distinct impact on leukemia hematopoiesis. While full-length PRDM16 suppresses the inflammatory pathway, its short-isoform exhibits the opposite effect in HSC [30]. On the other hand, PRDM16 lacking a PR domain triggers leukemic transformation in mice progenitor cells that carry a *p53* deletion. Hence, it is presumed that the long isoform of PRDM16 conceivably acts as a tumor suppressor [181]. The function of the PR domain in PRDM proteins during neuronal differentiation is unclear due to the small number of related studies, but the PR domain of PRDM16 is known to control epigenetic silencing determining the migration and position of neurons in the brain cortex [24]. In the same study, it was demonstrated that only a long version of PRDM16 is able to reverse the neuronal migration defects in PRDM16-deficient mouse brains [24]. Interestingly, the removal of a PR domain in the PRDM3/16 and FOG1/2 should also eliminate the NuRD domain, which is located at the N-terminus of the protein (Figure 4A). NuRD has shown paramount importance in the maintenance of hematopoietic stem cells [182] and neuronal development [183]. Therefore, it would be interesting to determine whether the effect of loss of PR domain results from the lack of interaction with cofactors such as the NuRD complex. 

## 9. CtBP Controls PRDM3, PRDM16, and FOG Function

CtBP controls the function of PRDM3, 16, and the FOG family through its specific binding sites [74,75,77,126]. CtBP1 and 2 are known as major transcriptional corepressors [184]. Ablation of CtBP proteins in early development is known to cause embryonic lethality [184], and information on the function of CtBP in the neural system is, therefore, limited. However, expression of CtBP1 has been reported in cultured hippocampal neurons, suggesting a potential function of CtBP1 in learning and memory [185]. Moreover, functional roles for CtBP in the neural system have been described in a number of studies in *D. melanogaster*. Drosophila CtBP (dCtBP) can control cell fate decisions in the sensory organ system as its loss of function leads to the formation of extra bristles, whereas flies with mutated dCtBP show a loss of bristles. Hamlet, fly PRDM3/16, loses its repressive activity when both CtBP domain and Zn fingers are deleted [103]. These results suggest that the function of PRDM3/16 and Hamlet is largely dependent on the CtBP protein, and their physical interaction is one of the key mechanisms by which PRDM3/16 orchestrates target gene expressions in stem cells and neuronal systems. 

Crosstalk between CtBP and sumoylation has been reported to be important for repressing transcriptional activity of PRDM16 protein [186]. Sumoylation is characterized by the reversible attachment of small ubiquitin-related modifier (SUMO) family members to lysine residue(s) located on target proteins via SUMO-activating enzyme subunit 1/2 (SAE1/2) and the SUMO-1-conjugating enzyme UBC9. The role of sumoylation has been studied in neurodegenerative diseases including Alzheimer’s disease [187,188]. *Sumo1*, *2*, and *3* are expressed in the developing mouse brain [189] and sumoylation acts as a modulator of several neural activities and of neuronal stem cell maintenance [190,191,192]. Among PRDM factors, PRDM3 and PRDM16 are known to be modulated by sumoylation. Sumoylation inhibits the DNA binding activity of PRDM3 to the *Bcl-xL* promoter, which eventually might be involved in mediating apoptosis [193]. Sumoylation of PRDM16 regulates its interaction with CtBP and activates PRDM16’s repressor activity. Moreover, the role of sumoylation in the regulation of PRDM16 has been well characterized in adipose tissue thermogenesis [194] and acute myeloid leukemia progression [195]. Thus, it has been proposed that sumoylation plays an important role in modulating PRDM3 and PRDM16 activity. Still, the role of sumoylation-dependent regulation of PRDM3 and PRDM16 in the nervous system remains to be elucidated. The new PRDM family member FOG1 is also regulated by sumoylation and specifically its interaction with CtBP. Interestingly, the sumoylation of FOG1 is modulated by the presence of phosphorylated residues in its sequence. Yang et al. [196] have proposed that several transcription factors possess a ψKxExxS/T (K = sumoylation target, S/T = phosphorylation target) motif which could be responsible for the interplay between sumoylation and phosphorylation marks on FOG1 activity. Thus, the interplay between post-translational modifications may orchestrate transcriptional repression via CtBP-dependent PRDM factors including PRDM3, 16, FOG1, and FOG2.

## 10. PRDM3/16 Function as Activators in Gene Regulation

Interestingly, PRDM3 and 16 interact not only with transcriptional repressors but also activators. Therefore, they are considered to be able to bidirectionally control gene expression (gene upregulation and downregulation) to exert their function. Acetylation of proteins acts as a positive regulator of transcription factors. PCAF and CBP acetylate PRDM3 [169,170], which enhances its transcriptional activity at the *GATA2* promoter (Figure 4C) [168]. Accordingly, the active PRDM3-GATA2 axis maintains a pool of hematopoietic stem cells. An interesting example of activation of gene expression by PRDM3 can be depicted by the interaction of PRDM3 with BRG1 in the embryonic fibroblast cell line (NIH 3T3). BRG1 significantly reduces the *E2F1* promoter activity, thus reducing the level of cell proliferation. By binding to BRG1, PRDM3 blocks this repression thereby accelerating the cell cycle [197]. BRG1 is a component of the SWI/SNF complex. SWI/SNF predominantly enhances gene expression by remodeling chromatin structure and making it more accessible to transcription factors but, depending on its protein partners, it may exhibit a repressive effect [198,199]. Interestingly, it has been shown that BRG1 can be also associate with the NuRD complex [200]. Therefore, the functional relationships between PRDM3, BRG1 and NuRD complex [73,197] remain to be clarified. Overexpression of C/EBPβ and PRDM16 reprograms murine and human fibroblasts into fat cells. It is suggested that C/EBP-β acts as a PRDM16 coactivator to enable the interaction of PGC-1α and PPARγ and activates cell differentiation [201]. It is known that C/EBP-β mediates the tuning of the transcription program to induce neurogenesis and to inhibit glial growth [202]. In mice, C/EBP-β has been shown to be involved in the survival and proliferation of neural stem cells in the hippocampus [203]. A similar effect was described by Shimada et al. where PRDM16 was indispensable for the maintenance of neural stem cells in the postnatal brain [31]. These similarities imply that C/EBP-β may also interact with PRDM16 during brain development and thus tune the genetic program in order to generate neuronal precursors.

Table 3 displays the most recognized cofactors interacting with PRDM3/16 and FOG1/2, and their roles.

## 11. Role of PRDM Proteins in Cancer Development and their Gene Mutations Found in Neuronal Diseases

Although PRDM proteins are recognized as regulators involved in cell differentiation [23], their commitment (suppressive or oncogenic) in the pathogenesis of human diseases such as carcinogenesis is also under investigation. PRDM1 is recognized as a tumor suppressor that inhibits the development of cancer cells, including lymphomas [232,233,234,235]. A chromosomal deletion or epigenetic silencing of PRDM1 expression is common in diffuse large B cell lymphoma subtypes [234,236,237]. Abnormal PRDM1 expression is also associated with other nonhematopoietic cancer cells, such as glioblastoma malignancies [238]. It has been identified that the downregulation of PRDM1 correlates with increased malignancy of lung tumors, where *PRDM1* disruption promoted neoplastic invasiveness [235]. The *PRDM*3 is a fused complex of two different transcripts, *MDS1* and *EVI1*. It is a frequent site of viral insertion and is associated with the development of myeloid leukemia [239,240]. PRDM14 overexpression, or retroviral integration in the gene locus, is often found in various types of cancer, and the molecular mechanism is set up to promote pluripotent traits [241]. PRDM14 overexpression is detected in approximately 25% of human lymphoid tumors. Mice bone marrow cells transduced with the PRDM14 expression vector often develop leukemia. The analysis of the gene expression profile indicated that *PRDM14* overexpressing cells showed significant enrichment of pluripotent genes and enhancement of the tumor-initiating pathway (WNT and RAS signaling) [242]. Although PRDM14 seems to be associated with the acquisition of an immortal phenotype, the neoplastic process driven by PRDM14 might depend on the tissue. Assessment of gene methylation levels from cervical scratches positive for human papillomavirus (HPV) at high risk of malignant disease showed an increased methylation level of the *PRDM14* gene [243]. It can, therefore, be assumed that PRDM14 acts as a tumor suppressor in cervical cancer. Hence, the role of PRDM14 in tumor development is enigmatic and requires further research. In lung cancer, the high methylation signature of the PRDM16 gene caused a significant reduction of its expression [244,245]. Another study indicated that relatively high expression of PRDM16 in patients with nonsmall cell lung cancer was associated with a preferable survival score [246,247,248]. At least in part, the PRDM16-mediated tumor inhibition could be explained by hammering the epithelial-to-mesenchymal transition in lung adenocarcinomas [246]. The role of FOG1 in the development of neoplasms is still ambiguous, albeit mutations within *FOG1* locus are extremely common (approximately 50% of cases) in patients with adrenocortical carcinoma [180]. Moreover, preliminary analysis indicates that *FOG1* is also frequently mutated in colorectal cancer [180]. Despite the small number of studies conducted on the relationship between FOG1 and initiation or tumor progression, the abovementioned findings indicate an unexplored phenomenon of the high frequency of *FOG1* mutations in tumors. FOG2, along with GATA4 and GATA6, is relatively highly expressed in sex cord-derived ovarian tumors [249]. Deregulation of the expression of *FOG2* and its cofactors has also been observed in ovarian granular cell tumors and ovarian stromal tumors in children [250,251]. Moreover, recent studies have shown that FOG2 is one of the most common mutated genes in the PRDM protein family. A high frequency of *FOG2* mutations is found in skin melanomas, uterine cancer, rectal cancer, esophageal cancer, gastric adenocarcinoma and lung tumors [180].

Human *PRDM* gene mutations in the nervous system are poorly recognized. Nevertheless, genetic abnormalities found within *PRDM* genes are significantly associated with neurological disabilities (Table 4). Clinical effects of *PRDM12* mutations in patients within the congenital insensitivity to pain are caused by defects in the development of nociceptors [91]. *FOG2* mutations were found to have a deleterious effect on brain structure development and patients exhibited a motor, linguistic and cognitive delay with seizure events [154,252].

## 12. Conclusions

Emerging evidence has suggested that PRDM factors cooperate with a number of protein partners to regulate a critical set of genes required for the maintenance of stem cell self-renewal and differentiation through multiple mechanisms. In this review, we proposed a NuRD and CtBP-dependent function of PRDM3/16 and FOG1/2 with respect to stem cell maintenance and neuronal differentiation. Moreover, we listed possible mechanisms of how these factors can regulate their target gene expression in a spatiotemporal and bidirectional manner. Although the PR domain that is contained in PRDM factors exerts methylation enzyme activity, our study suggests that cofactor-dependent regulation of PRDM3/16 and FOG1/2 is also one of the most important mechanisms to regulate PRDM factors function. Stem cell and neuronal cell fate are orchestrated by fine-tuned molecular mechanisms in which several transcription factors are encountered and dissociated. Furthermore, dysfunction of these factors causes abnormality in several tissues, and even leads to increased cancer risk. Therefore, identifying their stem cell and neuronal-specific cofactors will help to improve understanding of how they function in healthy and diseased conditions.

## Figures and Tables

**Table 1:** Roles of PRDM factors in stem cell system.

PRDM Protein	Role in Stem Cells	References
PRDM1/BLIMP-1	Expression level predicts embryonic stem cells and progenitors’ fate (mechanism partially dependent on PRL family members).	[34,35]
	Defines a mammary stem cell subpopulation with specific phenotype (mechanism unknown).	[39]
PRDM2/RIZ1	Maintains the key features of the quiescent state and affects the self-renewal of stem cells (interacts with the PRC2 complex and regulates the level of H3K9me2 within the promoter of *CCNA2A*).	[40,41]
PRDM3/EVI1	Inhibits a cell cycle and a differentiation of hematopoietic progenitor cells (indirectly upregulates genes related to keeping long-term hematopoietic stem cells like *Abca1*, *Cdkn1b*, and *Epcam*).	[42]
	Indispensable for intestinal stem cell formation during development (mechanism unknown).	[28,29]
	Induction of *Danio rerio* hematopoietic stem cell emergence by regulation of Notch pathway.	[43]
	Keeps long-term hematopoietic stem cell function during adult hematopoiesis by regulation of *Gata2*, *Sall2*, and *Pbx1* gene expression.	[44,45,46]
	Implicated in genesis of leukemia stem cells (precise mechanism unknown, putative target genes: *Gata1*, *Gata2*, *Mpl*, *Jag2*, *Setbp1*, and *Pbx1*).	[47,48]
PRDM4/PFM1	Regulates gene expression in embryonic stem cells mainly by binding proximally to transcription start sites of *Nodal* and *Klf5.*	[49]
	Controls the neural stem cells differentiation and proliferation by recruiting an arginine methyltransferase 5 (PRMT5).	[50]
PRDM5	Interacts with insulator proteins and modulates transcription program in embryonic stem cells.	[51]
	Transient expression with *Run1t1*, *Lmo2*, *Zfp37*, *Hlf* and *Pbx1* allows it to reprogram blood cells to hematopoietic cells.	[52]
PRDM11	Function unknown, expression in hematopoietic stem.	[53]
PRDM12	Overexpression stops cell proliferation in P19 cell line (direct mechanism unknown, upregulates p27 protein and increases the cell population in the G1 phase of the cell cycle).	[22]
PRDM14	Maintains pluripotency and self-renewal of embryonic stem (effect partially executed by recruiting repressive PRC2 complex and active DNA demethylation mediated via ten-eleven translocation (TET) proteins).	[36,54,55,56,57]
	Implicated in stem cell reprogramming (downregulates *Rnf12* gene expression via PRC2).	[58]
PRDM15	Maintains pluripotency of mouse embryonic stem cells by regulation of MAPK-ERK and WNT signaling.	[38]
PRDM16/MEL1	Supports maintenance of hematopoietic and neural stem cells (upregulates expression of *Hgf,* and *Foxj1* and regulates levels of reactive oxygen species).	[30,31,32,33]
FOG1/ZFPM-1	Increase erythropoietic differentiation rate in human hematopoietic stem cells (direct mechanism unknown, partially dependent on GATA-1, *c-myc*, and *c-myb* expression).	[59]
	Expressed in early hematopoietic cells in zebrafish and influences megakaryocytic and erythroid maturation.	[60]
	Interaction with NuRD promotes hematopoiesis.	[61]
	Required for the generation of erythroid- megakaryocytic progenitors in mice (putative mechanism addressed to Trib2-dependent C/EBPα and C/EBPβ degradation).	[62]
FOG2/ZFPM-2	Human bone marrow mesenchymal stem cells with high FOG2 expression display cardiomyogenic potency (mechanism unknown).	[63]

**Table 2:** The roles of PRDM factors in the neuronal system.

PRDM Protein	Nervous System Function	References
PRDM1/BLIMP-1	Leads to specialization and identity of photosensory neurons (directly reduces *Chx10* expression).	[94,95,96,97]
PRDM2	Monomethylates H3K9 in neurons of the rat dorsomedial prefrontal cortex and is involved in alcohol dependence.	[98]
PRDM3/EVI1	*Caenorhabditis elegans* egl-43 protein (ortholog of PRDM3) is required for the proper development of phasmid neurons (mechanism unknown).	[99]
	Knock-out mice exhibit malformation of neuronal development during mouse embryo growth (mechanism unknown).	[100]
	The overexpression of *Prdm3* triggers neurogenesis in P19 cell line (direct mechanism unknown, high expression of *Mash1*, *Ngn1*, *NeuroD1* observed).	[101]
	Gene knock-out leads to precocious neuronal differentiation in the P19 cells (direct mechanism unknown, increased expression of MAP2 and β-III TUBULIN).	[102]
	Hamlet (*Drosophila melanogaster* PRDM3 and PRDM16 homolog) removes notch-dependent fate signature during neuronal-class diversification via direct chromatin-modification.	[103]
	Regulates homeostatic synaptic plasticity by downregulation of miR-124.	[104]
PRDM4	Controls neural stem cell proliferation and differentiation by protein arginine methyltransferase 5 (PRMT5).	[50]
PRDM5	Enhances neuronal apoptosis triggered by lipopolysaccharide (direct mechanism unknown).	[105]
	Low expression associated with neurotherapeutic effects of miR-182/7a in spinal cord injury (SCI) model.	[106]
	Overexpression increases abnormalities mediated by WNT signaling during the development of anterior neural structures in *Danio rerio.*	[107]
PRDM8	Along with BHLHB5 creates a transcriptional repressor complex required for normal development of specific neural circuits.	[90]
	Regulates promoter activity of *Prkca* and thus retinal bipolar cell development and survival.	[108]
	Controls the morphological changes at the multipolar phase during neocortex development by indirect repression of guidance molecules, like EPHA6, NRP2, and EBF3.	[89]
	Gene knock-out impairs development of neocortical neurons (direct mechanism unknown, deregulation of *Fgf5*, *Hmcn1*, *Antxr2,* and *Slc15a2* gene expression).	[26]
PRDM12	Orchestrates sensory neuron development and specification in part by dimethylation of H3K9 (target genes unknown).	[91,109]
PRDM13	Generates neuronal specification by repression of bHLH transcriptional activators.	[110]
	Inhibits glutamatergic and promotes GABAergic neuronal development in the neural tube by repressing *Ascl1* activation of *Tlx3* gene expression.	[71,111]
PRDM14	Regulates axon growth of primary motoneurons in *Danio rerio* by regulation of *islet2* promoter activity.	[112]
PRDM15	Gene knock-out causes brain malformations via deregulation of NOTCH- and WNT- dependent pathway.	[113]
PRDM16/MEL1	Coordinates neuronal-dependent brain vascularization via SMOC1 protein.	[114]
	Involved in cortical neuron migration and positioning in part by repressing PDZRN3 expression.	[24,89]
FOG2/ZFPM-2	Controls axonal targeting and differentiation of corticothalamic projection neurons (by interaction with COUPTF1, GATA2, and GATA4 to reduce *Citp2* expression).	[93]
	Together with GATA4 and GATA6 increases *Kv4.2* gene (a subunit of somatodendritic A-type potassium channels) expression in PC12 neuron-like cell line.	[115]

**Table 3:** Summary of PRDM3/16 and FOG1/2 interaction with their known cofactors. The PRDM3/16 and FOG1/2 proteins affect the genetic program and cell functions by interaction with their cofactors. This influence may have a positive or negative effect.

Gene Symbol	Interacting Protein	Gene Repression	Refs
PRDM3	CtBP1/2	Increases proliferation of Mv1Lu cell line and (murine hematopoietic precursor cell line) 32Dc13.	[79,168,204,205,206,207]
	RUNX1	Blocks the differentiation of 32Dcl3 cells and induce cell death.	[208]
	GATA1	Represses of erythroid-lineage differentiation in murine bone marrow cells.	[209]
	PU.1	Impairs myelopoiesis in bone marrow progenitors.	[210]
	SMAD3	Increase the growth of myeloid cells.	[211]
	JNK	Stops stress-induced cell death in NIH 3T3 cells.	[212]
	SNAIL, HDAC1	Fosters epithelial-to-mesenchymal transition in nasopharyngeal carcinoma cell line (6-10B cells).	[213]
	DNMT3A/B	Represses regulatory regions of miR-124-3, function unknown.	[214]
	SUV39H1, G9a	Bone marrow immortalization and transcription suppression.	[215,216]
	HDAC1	Stops the homeostatic response in cortical neurons.	[56,104,204]
	NuRD complex (RBBP4)	Function unknown.	[73,204]
	PRC2 complex (EZH2, SUZ12, EED)	Myeloid transformation of bone marrow.	[15]
	HIC1	Abolishes the PRDM3-mediated inhibition of apoptosis in HCT116 cells.	[217]
	p65	Represses inflammation via inhibition of NF-κB in middle ear epithelial and airway epithelial cells.	[218]
	P/CAF and CBP	Cell function unknown, changes nuclear localization pattern of PRDM3.	[170]
	BRG1	Increases proliferation of 32Dcl3 cells (murine hematopoietic precursor cell line).	[197]
	P/CAF	Increases proliferation and maintenance of HSC.	[168]
	NuRD complex (MBD3)	Hinders the histone deacetylation activity of NuRD (in vivo function unknown).	[219]
PRDM16	CtBP1/2	Reduces gene expression involved in white fat development.	[74]
	UBC9	Mediates CtBP1/2-dependent blocking of myeloid differentiation of L-G3 cells.	[186]
	EHMT1	Blocks myogenic differentiation.	[220]
	SMAD3	Inhibits the cytostasis in MKN28 gastric cancer cells.	[221]
	LSD1	Diminishes the expression of white fat genes.	[222]
	NuRD complex (RBBP4)	Function unknown.	[73]
	C/EBPβ	Sets up a transformation of myoblastic precursors into brown fat cells.	[201]
	PPARγ	Stimulates a brown adipogenesis.	[119]
	MEDIATOR complex (MED1)	Adjusts a chromatin architecture in key brown fat genes.	[223]
	ZFP516	Promotes brown fat development in white fat cells.	[224]
	PGC-1α	Highly stimulates a transcriptional program of brown fat development.	[74]
FOG1	GATA1	Represses erythroid cell maturation.	[135,225,226]
	NuRD complex (MTA1/2, p66, RBBP4)	Inhibits GATA-1-dependent gene transcription involved in the terminal erythroid maturation.	[128]
	CtBP1/2	Hampers erythropoiesis in *Xenopus.*	[77,227]
	LSD1	Function unknown.	[228]
FOG2	COUP-TF2, COUP-TF3	Specific function unknown, probably involved in cardiac morphogenesis.	[229]
	Art27	Boosts the transcriptional repression of GATA4 and thus gene expression involved in cardiac development.	[230]
	CtBP1/2	Suppresses the adipogenesis of 3T3-L1 cells.	[126]
	NuRD complex (MTA1/RBBP4/7)	Increases cardiomyocyte proliferation mediated via repression of *Cdkn1a* transcription.	[76,167]
	GATA4	Decreases *α-MHC* promoter in primary cardiomyocytes.	[147]
	RXRα	Boosts the transcriptional repression of GATA4 and thus gene expression involved in cardiac development.	[231]
	GATA6	Function unknown.	[172]
	GATA4	Increases the α-MHC promoter activity in Cos cells.	[147]

**Table 4:** Clinical effect of mutation in PRDM genes within the neurological system.

Gene	Mutation Type	Phenotype	References
*PRDM8*	Homozygous missense mutation	Progressive myoclonus epilepsy.	[253]
*PRDM12*	Several mutations within gene, autosomal recessive	Neuropathy, congenital insensitivity to pain.	[91]
*PRDM13*	Heterozygous mutation, tandem duplication	Macular dystrophy, North Carolina type.	[254]
*PRDM16*	Chromosome 1p36 deletion	Mental retardation.	[255]
*FOG2*	46XY sex reversal 9	Subtentorial ventricular dilation, major learning and reading difficulties.	[252]
	46,XY,del (1) (q41q42.12)	Atrophy of the right hippocampus, loss of volume in the right side of the brain, seizures.	[154]
